# Modulation of Redox Balance by Phytochemicals: Implications for Cardiovascular Health

**DOI:** 10.3390/nu18081204

**Published:** 2026-04-10

**Authors:** Morana Jaganjac, Nelson N. Orie

**Affiliations:** 1 Laboratory for Oxidative Stress, Division of Molecular Medicine, Rudjer Boskovic Institute, Bijenicka 54, 10000 Zagreb, Croatia; 2 Division of Medicine, University College London, London NW3 2PF, UK

**Keywords:** oxidative stress, vascular inflammation, endothelial dysfunction, redox homeostasis, phytochemicals, cardiac protection, vascular protection

## Abstract

Cardiovascular diseases (CVDs) are the leading cause of mortality worldwide, with oxidative stress playing a major role in disease pathogenesis by promoting endothelial dysfunction, vascular inflammation, and tissue damage. Oxidative stress results from an imbalance between antioxidant defenses and reactive oxygen species (ROS) in favor of ROS. Excessive ROS damage macromolecules and may trigger a chain reaction of lipid peroxidation, protein modification, and DNA damage. Phytochemicals are naturally occurring compounds in fruits and vegetables that may modulate redox homeostasis and positively impact cardiovascular health. The flavonoid Quercetin, Resveratrol, Curcuminoids, Coenzyme Q10, Hydroxysafflor yellow A, and Vitamins C and E have shown promise in human studies for improving endothelial function, lipid profile and markers of oxidative stress and inflammation. Among the key mechanisms of protection are their antioxidant role, anti-inflammatory role or modulation of nuclear factor erythroid 2-related factor 2 (Nrf2) pathway, all of which contribute to cardiovascular protection. However, there are challenges associated with their use for health, such as the complexity of their quality and quantity, which require standardization, as well as their mechanisms of effects. Moreover, their systemic availability and bioactivity largely depend on metabolic transformation by the host gut microbiota. This review analyzed relevant publications in PubMed, Scopus, and Web of Science, up to February 2026, and summarizes current knowledge on phyto–chemical-mediated modulation of oxidative stress and its implications for cardiovascular protection in humans. The evidence suggests that phytochemicals hold promise for CVD prevention and treatment, but more work is needed to achieve standardization in quality and quantity.

## 1. Introduction

Cardiovascular diseases (CVDs) are among the leading global causes of mortality, accounting for approximately 17.9 million deaths annually, or 32% of all deaths worldwide [1,2]. The increasing incidence of atherosclerosis, hypertension, and heart failure highlights the urgent need for prevention strategies that complement current medical approaches. Thus, dietary interventions have gained substantial attention due to their beneficial effects in modulating diverse risk factors associated with CVD.

Cardiovascular tissues are particularly susceptible to excessive reactive oxygen species (ROS) and reactive nitrogen species [3,4,5]. ROS, generated during physiological and pathological processes, disrupt cellular homeostasis and induce damage to DNA, proteins, and lipids, trigger lipid peroxidation in the vascular wall, or may activate pro-inflammatory pathways such as nuclear factor kappa B (NF-κB), all of which support the development and progression of atherosclerosis [6]. Phytochemicals were thought to act predominantly as direct antioxidants capable of scavenging free radicals in vitro [7]. However, pharmacokinetic studies have demonstrated that their plasma concentrations after ingestion typically fall within the low micromolar to nanomolar range, which makes their direct effect biologically unlikely [8,9]. Current evidence instead characterizes phytochemicals as modulators of intracellular signaling pathways that indirectly influence redox balance. However, despite growing interest in phytochemical-based cardiovascular protection, the literature remains fragmented across antioxidant, anti-inflammatory, and nutritional perspectives, without sufficient integration of these processes into a unified cardiovascular redox framework. In particular, the mechanistic links between oxidative stress, lipid peroxidation, inflammatory signaling, endothelial dysfunction, and vascular injury remain insufficiently synthesized in the context of phytochemical action.

Furthermore, their bioactivity is closely linked to the gut microbiota, which metabolizes dietary phytochemicals into diverse bioactive derivatives with distinct physiological effects [10,11,12]. This review aims to provide an integrated and up-to-date overview of the mechanisms through which major phytochemical classes interact with cellular redox regulatory systems relevant to cardiovascular health.

Relevant publications for this narrative review were collected through searches of PubMed, Scopus, and Web of Science covering the literature published up to February 2026. The research included combinations of the following terms: phytochemicals, polyphenols, flavonoids, carotenoids, oxidative stress, redox signaling, lipid peroxidation, Nrf2, NF-κB, inflammation, endothelial dysfunction, and cardiovascular disease. Emphasis was placed on studies with direct relevance to human cardiovascular biology, including experimental and clinical investigations, together with selected review articles addressing the mechanistic links between oxidative stress, inflammation, and phytochemical-mediated cardioprotection.

## 2. Phytochemicals

Phytochemicals are secondary metabolites produced by plants, often playing roles in defense or providing pigmentation. Within cardiovascular health research, particular attention is given to polyphenols, especially flavonoids and lipid-soluble carotenoids.

**Figure 1:**
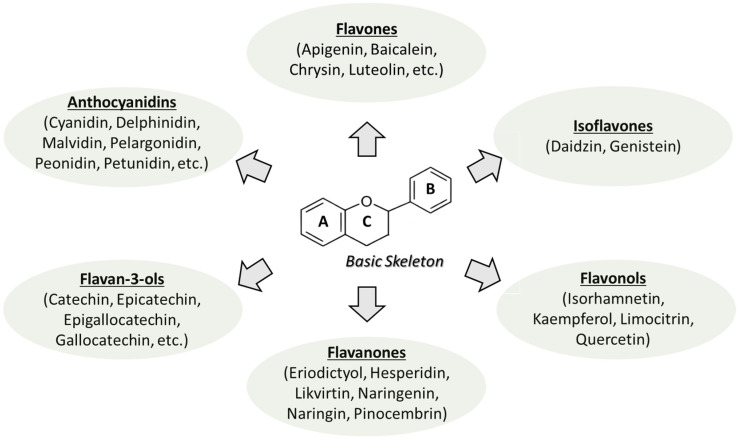
Flavonoid backbone is composed of two benzene rings (A and B) linked through a three-carbon unit that commonly forms an oxygen-containing heterocyclic C-ring. Structural variation within the C-ring underlies the major flavonoid subclasses.

Polyphenols are defined by the presence of multiple phenolic hydroxyl groups attached to one or more aromatic rings, which are responsible for their chemical reactivity and biological activity. Polyphenols are categorized into several groups, with flavonoids representing the most abundant and extensively studied group of polyphenols. Figure 1 illustrates a conserved C_6_–C_3_–C_6_ flavonoid backbone consisting of two benzene rings (A and B) connected via a three-carbon unit that typically forms an oxygen-containing heterocyclic C-ring [13]. Based on structural variations in the C-ring, flavonoids are further classified into several major subclasses and includes flavones, flavanones (dihydroflavones), isoflavones, flavonols (3-hydroxyflavone), flavan-3-ols (dihydroflavonols/catechins) and anthocyanins [14]. In addition to flavonoids, non-flavonoid polyphenols constitute several important subclasses of bioactive compounds, such are phenolic acids, stilbenes and lignans. Main flavonoid and non-flavonoid subclasses, as well as their representative compounds, dietary sources, and functional characteristics, are presented in Table 1.

Carotenoids are structurally different from polyphenols and consist of lipid-soluble tetraterpenoid pigments responsible for the yellow, orange, and red color of plant [29]. They are hydrophobic and may thus be incorporated into cellular and organelle membranes [30]. Carotenoids may prevent lipid peroxidation of membrane-bound polyunsaturated fatty acids, which is one of the critical steps in the early development of atherosclerosis. Key dietary carotenoids include β-carotene, lutein, zeaxanthin and lycopene [31]. Among those, lycopene was shown to significantly reduce oxidative stress, improve flow-mediated dilation, and decrease the incidence of myocardial infarction in human cohort studies [32,33].

## 3. Antioxidant Activity of Phytochemicals

Phytochemicals may act as antioxidants via either direct or indirect mechanisms. Flavonoids, polyphenols, and carotenoids may scavenge ROS, efficiently neutralizing free radicals, and thus preventing oxidative injury to lipids, proteins, and nucleic acids [34,35]. Complementing this scavenging capacity is their ability to inhibit ROS-generating enzymes such as NADPH oxidase, as well as to chelate redox-active metals that otherwise catalyze deleterious oxidative reactions [36,37].

In addition, phytochemicals may also enhance the activity of endogenous antioxidant enzymes such are superoxide dismutase, catalase, and glutathione peroxidase, thus reinforcing cellular defenses against oxidative stress [38,39]. Their role is crucial in maintaining vascular function and mitigating ROS-driven tissue damage. Importantly, this indirect antioxidant activity is not limited to enzyme support at the functional level but also involves transcriptional upregulation of antioxidant and cytoprotective genes through redox-sensitive signaling pathways. In this context, Nrf2 represents a central regulatory mechanism through which many phytochemicals enhance endogenous cellular defense capacity and contribute to cardiovascular protection [40]. Thus, in addition to acting as radical scavengers, phytochemicals may exert antioxidant effects by activating adaptive stress–response pathways that preserve redox homeostasis and cellular resilience. A more detailed discussion of phytochemicals as modulators of Nrf2 function is provided in Section 4. Some phytochemicals, such as betanin, induce transcription of genes encoding antioxidant and cytoprotective proteins while simultaneously suppressing prooxidant and inflammatory pathways [41].

By limiting oxidative stress and the propagation of lipid peroxidation, phytochemicals may reduce the formation of reactive lipid-derived aldehydes and other electrophilic products that can impair membrane integrity, mitochondrial function, and cellular homeostasis promoting diverse pathologies [3,42,43,44]. This aspect is particularly important because lipid peroxidation products are not only markers of oxidative injury, but may also function as bioactive mediators that amplify inflammatory signaling and vascular dysfunction [3,45,46]. Thus, the antioxidant activity of phytochemicals may be relevant not only for limiting molecular damage, but also for interrupting redox-driven inflammatory propagation in cardiovascular tissues. At the same time, controlled modulation of cellular redox balance by phytochemicals may support physiological redox signaling pathways that regulate adaptation to stress and cell survival or even regeneration [46,47]. These properties highlight the importance of phytochemicals in not only preventing oxidative damage but also in maintaining balanced redox regulation under physiological and pathological conditions.

## 4. Phytochemicals as Modulators of Nrf2 Function

As outlined in Section 3, a substantial part of the antioxidant activity of phytochemicals is mediated indirectly through the activation of endogenous defense pathways, among which Nrf2 signaling represents one of the central mechanisms. Recent evidence further supports the concept that activation of Nrf2 signaling is a major mechanism by which natural products protect against oxidative and inflammatory cardiovascular injury [40]. The Nrf2 pathway is a master redox regulator and is involved in cellular protection against oxidative stress [4]. Under basal conditions, Nrf2 is sequestered in the cytoplasm by Kelch-like ECH-associated protein 1 (Keap1) and targeted for ubiquitination. Upon activation, Nrf2 dissociates from Keap1 and translocates to the nucleus. Flavonoids and related polyphenols are among the most potent natural activators of Nrf2, and may promote nuclear translocation of Nrf2, induce the transcription of antioxidant enzymes, modulate upstream kinases, or prevent Nrf2 ubiquitination by either inhibiting Keap1 or disrupting Keap1–Nrf2 interactions [48,49,50]. Activation of Nrf2 promotes transcription of an array of cytoprotective genes, including heme oxygenase-1 (HO-1), that mitigates ROS-induced damage and attenuates inflammation, thus contributing to cardioprotection [51]. A detailed schematic representation of the Keap1-Nrf2-ARE activation cascade and its downstream cytoprotective consequences have been published elsewhere and is therefore not reproduced here [4]. In addition, Nrf2 activation may indirectly restrain redox-sensitive inflammatory pathways such as NF-κB, further supporting the concept that phytochemicals act on an integrated redox–inflammatory network rather than on oxidative damage alone. Recent reviews have emphasized Nrf2 signaling as a major mechanism by which natural products protect against oxidative and inflammatory cardiovascular injury [40].

Lycopene can activate Nrf2, leading to the upregulation of antioxidant enzymes including HO-1 and NAD(P)H quinone oxidoreductase 1 [52]. Tiliroside and quercetin hinder Keap1-mediated Nrf2 ubiquitination, thereby stabilizing the Nrf2 and promoting its nuclear accumulation [50,53]. Quercetin binds competitively to the Arg483 site of Keap1, preventing Nrf2 sequestration [53]. Indirect activation of Nrf2 is achieved through kinase-mediated signaling modulated by phytochemicals, such as genistein [54]. Among the kinases, AMP-activated protein kinase (AMPK), mitogen-activated protein kinases (MAPK), and protein kinase C (PKC) pathways are known to phosphorylate and activate Nrf2 [54,55,56]. Norartocarpin, an isopentenyl-substituted flavonoid, enhances Nrf2 protein stability and facilitates nuclear translocation by activating multiple kinases, including MAPK, PI3K, PKC, and protein kinase R-like endoplasmic reticulum kinase (PERK) [56]. Procyanidin B2 promotes Nrf2 translocation via the ERK and p38-MAPK pathways, enhancing the expression of glutathione S-transferase P1 [57]. Furthermore, some phytochemicals, such as lycopene or curcumin, may trigger autophagic degradation of Keap1 via p62, further amplifying Nrf2-induced transcription [58,59].

Kinase pathways influenced by flavonoids and their role in Nrf2 activation are listed in Table 2.

Beyond direct kinase-mediated activation, flavonoids regulate Nrf2 through epigenetic modifications. These include DNA demethylation and histone acetylation, which enhance transcription. DNA methylation of the Nrf2 promoter represses its transcription. For example, luteolin and delphinidin decrease CpG methylation, increasing Nrf2 mRNA expression and activation of downstream antioxidant genes [60,61]. Moreover, some phytochemicals such as sulforaphane can inhibit histone deacetylases promoting histone acetylation and epigenetic reactivation of Nrf2 [62]. Beyond antioxidant gene induction, Nrf2 activation may also indirectly attenuate inflammatory signaling, highlighting its importance at the interface between redox regulation and vascular inflammation.

## 5. Phytochemical Modulation of Inflammation

Inflammation is the body’s response to injuries involving a complex set of defense mechanisms working together to restore normal state. This often involves local release of histamine, prostaglandins, and nitric oxide (NO), all of which contribute to increased blood flow and immune cell infiltration into the affected area [63]. Pro-inflammatory cytokines such as tumor necrosis factor-alpha (TNF-α), interleukin-1 (IL-1) and interleukin-6 (IL-6), which are secreted by the activated immune cells, stimulate increased expression of leukocyte adhesion molecules on endothelial cells to promote increased permeability of leukocytes [64]. In the short-term, inflammation can lead to the restoration of homeostasis and tissue repair [65]. When that fails, serious tissue dysfunction becomes a possibility.

Plant-derived compounds are increasingly being researched as natural alternatives for the treatment of various diseases including inflammatory conditions. This is because of their demonstrated potential as anti-inflammatory compounds in numerous studies (in vitro and in vivo). Phytochemicals may influence cardiovascular pathology not only through antioxidant effects, but also by modulating inflammatory mediators and signaling pathways, including COX-related pathways, TNF-α-associated responses, and AMPK-dependent mechanisms [66]. There is some evidence to suggest that phytochemicals have the potential to inhibit the expression of pro-inflammatory genes such as NF-κB, COX-2 and iNOS [67] and to block the synthesis of inflammatory cytokines [68]. Some of the phytochemicals with these properties include resveratrol, quercetin, curcumin, genistein epigallocatechin-3-gallate, apigenin and anthocyanins (Table 3). Resveratrol (a phenolic stilbene derivative) found in grape skins, berries and peanuts, is a potent Nrf2 activator [4]. It has also been shown to block NF-κB activation [69] and to reduce the synthesis of NF-κB-induced pro-inflammatory cytokines, including TNF-α, IL-6, and IL-1β, and the free radicals, NO and ROS [70]. Its effect might also be epigenetic. It is reported to downregulate micro RNA (miR)-155 and miR-21 and to suppress miR-146a by its anti-inflammatory and antioxidant activities [71,72]. Quercetin, which is found in citrus fruits, apples, onions, red grapes and tea [73], has been shown to have the potential to prevent NF-κB translocation and to inhibit cyclooxygenase-2 (COX-2) and inducible nitric oxide synthase expression in macrophages and human peripheral blood mononuclear cell [67]. It also appears to activate Nrf2, which in turn inhibits the NF-κB pathway [74,75]. Like resveratrol, quercetin also decreases miR-155 expression [74,76]. Curcumin, which is found in turmeric, is reported to activate Nrf2 and as such, it blocks NF-κB activation [77], inhibits lipid peroxide formation and reduces oxidative stress [78,79]. It also downregulates miR-155 [80]. Genistein is an iso-flavonoid found in soy-based foods, red clover and legume. Its anti-inflammatory property is based on its reported ability to activate Nrf2, block NF-κB-induced damage and downregulate IL-6 and intercellular adhesion molecule-1 expressions [81]. Like resveratrol, quercetin and curcumin, it can also suppress miR-155 [82]. Another phytochemical that is reported to suppress inflammation by activating the Nrf2 pathway and decreasing NF-κB nuclear translocation [83] is Epigallocatechin-3-Gallate. This anthraquinone compound is abundant in buckthorn, knotweed and rhubarb [84]. Apigenin is a plant-derived flavonoid abundant in many fruits and vegetables, including parsley, celery, and chamomile tea [85]. It is reported to exert its anti-inflammatory effects through the inhibition of COX-2 and NF-κB, and the downregulation of the Toll-like receptor 4 (TLR4)/NF-κB signaling pathway [4,86]. It can also downregulate miR-155 by inhibiting NF-κB [87]. Anthocyanins are flavonoids found in berries, grapes, and potatoes and reported to ameliorate neuroinflammation by decreasing TLR4 expression, activating the Nrf2/HO-1 signaling pathway and inactivating NF-κB [88]. The polyphenolic compound ellagic acid which is found in fruits, including raspberries and strawberries, mushrooms, and nuts, can reduce inflammatory responses and oxidative stress by inhibiting TLR4, activating Nrf2 and reducing the activity of NF-κB [89]. Terpenoids such as Tanshinone IIA, Carvacrol Thymol and Boswellic acids also activate Nrf2 expression and reduce miR-155 expression [90,91]. Isothiocyanates such as Sulforaphane found in broccoli, cabbage, cauliflower, and kale and allyl-Isothiocyanate found in mustard, wasabi, and horseradish [74] are also reported to activate the Nrf2 pathway, and to reduce NF-κB expression [92]. A summary of the reported mode of anti-inflammatory activities of the reviewed compounds is presented in Table 3.

The mechanisms described above clearly demonstrate the anti-inflammatory potential of these phytochemicals. However, their bioavailability when consumed in plant-based diets is relatively low to produce the outline biological effects in humans [93]. It is therefore important to find safe ways to boost their bioavailability to fully benefit from their apparently rich therapeutic potential. A few attractive suggestions have been made to increase their bioavailability, such as preparing them as concentrates without destroying their natural state, improving formulations, e.g., liposomal phytochemicals, phytochemical nanoparticles, and phytochemicals–phospholipid complex [94], and co-administration with other treatments. For instance, the co-administration of curcumin with reinstate (Histone Deacetylase inhibitor) ameliorated antibody-dependent neurodegeneration compared with the use of reinstate alone [95], and the co-administration of resveratrol with metformin (anti-diabetic drug) successfully reduced inflammation in diabetic mice [96]. An understanding of the mechanisms of such synergism will significantly help to boost the therapeutic utility of phytochemicals for the treatment of inflammatory diseases.

## 6. Cardiac and Vascular Protective Activity

The World Health Organization estimates that 17.9 million people die each year from cardiovascular diseases (CVDs), which include coronary heart disease, cerebrovascular disease, peripheral arterial disease, and other disorders of the heart and blood vessels [97]. In 2022, these accounted for about 32% of all deaths globally, with more than four out of five of these deaths due to heart attacks and strokes in people under the age of 70 [98].

At the mechanistic level, oxidative stress and inflammation should be viewed as mutually reinforcing processes in cardiovascular pathology. Excess ROS may activate redox-sensitive inflammatory pathways, including NF-κB signaling, increase cytokine production, and promote endothelial dysfunction, while inflammatory mediators further enhance ROS generation and lipid peroxidation [3,40,66]. Phytochemicals may therefore exert cardiovascular protection not only by attenuating oxidative damage, but also by modulating inflammatory targets and restoring redox–inflammatory balance. Vascular inflammation and oxidative stress are major drivers of the risk factors for CVD such as hypertension, endothelial dysfunction, dyslipidaemia and atherosclerosis [99]. Although inflammation is a normal biological response of the body to tissue damage, if unresolved, it leads to the overproduction of cytokines and ROS, which can exacerbate the tissue damage associated with CVDs [100]. Inflammation can also provoke abnormal lipid metabolism and cholesterol accumulation which can cause endothelial dysfunction and promote atherosclerosis [101]. Both systemic and local myocardial inflammation can induce an increase in the release of ROS and oxidative stress, which in turn can trigger further inflammation and endothelial dysfunction. Oxidative stress can also directly impair the function of cardiac muscle cells, leading to cardiac dysfunction and potentially heart failure [102].

**Figure 2:**
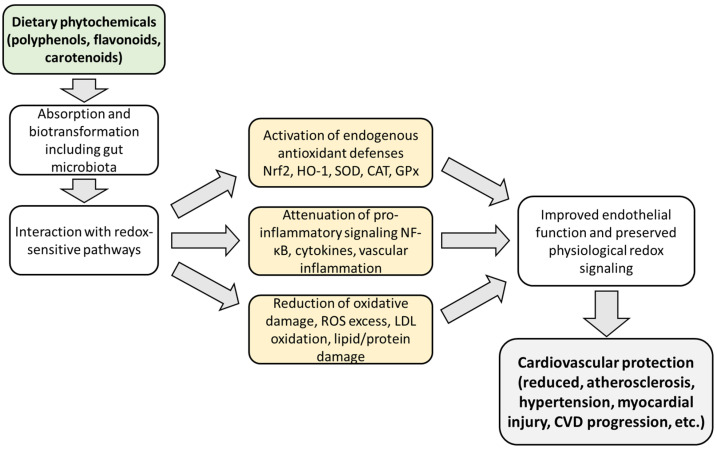
Simplified scheme of the mechanisms by which phytochemicals may support cardiovascular health. After absorption and biotransformation, including metabolism by the gut microbiota, phytochemicals and their metabolites interact with redox-sensitive signaling pathways. This may lead to activation of endogenous antioxidant defenses, attenuation of pro-inflammatory signaling, reduction in oxidative damage, preservation of physiological redox signaling, and improvement in endothelial function, ultimately contributing to cardiovascular protection. Abbreviations: CAT, catalase; CVD, cardiovascular disease; GPx, glutathione peroxidase; HO-1, heme oxygenase-1; LDL, low-density lipoprotein; Nrf2, nuclear factor erythroid 2-related factor 2; ROS, reactive oxygen species.

The literature is full of publications that demonstrate the potential of phytochemicals to prevent or reverse the pathophysiology of CVDs based on their anti-inflammatory and antioxidant properties. A simplified overview of the main mechanisms by which phytochemicals may contribute to cardiovascular protection is presented in Figure 2.

Most available studies were carried out in vitro or in animals and although they indicate potential, they face the challenge of translation and utility in humans. The current review focuses on studies in humans or with human samples to highlight progress made as well as challenges that remain in the therapeutic utility of phytochemicals for the prevention and treatment of CVD in humans. In one of the studies, the effect of intake of dietary flavonoids on the risk factors for chronic diseases was investigated in 805 men (65–84 years), who were followed for up to five years [103]. The results showed that high intake of flavonoids predicted lower mortality from coronary heart disease, which was highly significant. The predominant flavonoid in the food consumed by the participants was quercetin which intake gave essentially the same relative risks as tertiles of total flavonoid intake in the study. The study concluded that flavonoids in regularly consumed foods may reduce the risk of death from coronary heart disease in elderly men. This was confirmed by subsequent studies that showed that dietary flavonoid intake was inversely associated with coronary heart disease in humans [104,105]. More so, oral administration of quercetin (500 mg/day, for eight weeks) post-myocardial infarction (MI) increased total antioxidant capacity of the patients [106].

One of the most studied phytochemicals for its cardioprotective potential is resveratrol, which is found in wine, peanuts, pistachios (*Pistacia vera*) and *Vaccinium* spp. berries. A triple-blind, placebo-controlled clinical trial in patients with stable coronary artery disease concluded that resveratrol has cardiovascular benefits, which they suggested was due to increased serum adiponectin, decreased thrombogenic plasminogen activator inhibitor type 1 and inhibition of atherogenic signals in blood mononuclear cells in these patients [107]. Although its effect on cholesterol accumulation is still debatable, a clinical study of 62 patients with type 2 diabetes demonstrated that resveratrol supplementation of 250 mg/day for three months was associated with decreased total cholesterol, although no significant changes in high-density lipoprotein (HDL) and low-density lipoprotein (LDL) were observed [108]. In another clinical study, supplementation with a grape extract containing resveratrol for six months resulted in decreased level of oxidized LDL and apolipoprotein B in patients undergoing primary prevention of cardiovascular disease [109]. In addition to dyslipidaemia, high blood pressure is also a major risk factor for CVD [110]. Resveratrol treatment at high doses (≥150 mg/d) significantly decreased systolic blood pressure in patients with type 2 diabetes [108,111,112] and increased flow-mediated dilatation in both overweight/obese men and post-menopausal women with untreated borderline hypertension [113], consistent with improving endothelial function. This improvement was attributed to increased NO signaling and stimulation of Ca^2+^-activated K^+^ channels [114]. These results suggest that resveratrol’s efficacy is dependent on factors such as dose, disease type, patient’s weight and the duration of treatment.

In another study, Ozdemir et al. [115] investigated the effects of the aqueous extract of *Origanum onites*, which contains a wide array of flavonoids, on the endothelial function and antioxidant status of 48 patients with mild hyperlipidaemia who did not require drug therapy. The control group (16 patients) received lifestyle and low-fat dietary advice while the study group (32 patients) were prescribed 25 mL of aqueous distillate of *Origanum onites*, in addition to lifestyle and low-fat dietary advice, for three months. At the end of the three months, HDL-cholesterol was significantly increased and LDL-cholesterol and high-sensitivity C-reactive protein were significantly decreased in the study group compared with the control group. Flow-mediated dilatation of the brachial artery, which reflects endothelial function, was also improved in the study group. The results suggested that the contents of this plant extract have the potential to lower vascular inflammation and endothelial dysfunction which in turn would lower the risk of atherosclerosis in these patients.

Curcuminoids (4 g/day) lowered the levels of malondialdehyde, C-reactive protein and *N*-terminal pro-B-type natriuretic peptide in 121 patients undergoing coronary artery bypass grafting [116], thereby reducing the risk of myocardial infarction in these patients. It has also been shown to reduce ischaemia–reperfusion-induced mitochondrial oxidative damage via the activation of silent information regulator 1 signaling [117,118]. However, its effect on lipid profile in obese individuals appears to be limited to reduction in serum triglycerides with no effect on other lipid profile parameters [119,120].

The other phytochemicals that have shown potential to benefit patients with CVD are Coenzyme Q10 (CoQ10) and safflower yellow A. CoQ10 is found in nuts and made in the body [121]. When supplemented at a dosage of 150 mg/day, it decreased the inflammatory marker IL-6 in patients with coronary artery disease [122,123]. In another study, CoQ10 supplementation was associated with improved blood pressure, serum HDL-C as well as LDL-C/HDL-C and TC/HDL-C ratios in patients with myocardial infarction and hyperlipidaemia [124]. On its side, Hydroxysafflor yellow A found in Safflower (*Carthamus tinctorius* L.) improved clinical symptoms of unstable angina in combined therapy [125] and in patients with acute ischemic stroke and blood stasis syndrome [126].

The antioxidant properties of vitamins C, E, or their combinations, have often been explored in the management of CVDs. It is worth noting that all steady state comparative bioavailability studies in humans have shown no differences between synthetic and natural vitamin C, regardless of the subject population, study design or intervention used. Even where pharmacokinetic studies have shown transient and small differences, such differences are likely to have minimal physiological impact [127]. Vitamin C appears to be particularly effective in hypertension which is associated with reduction in baroreflex sensitivity due to oxidative stress. Acute infusion of vitamin C into 32 patients with essential hypertension and 20 normotensive controls significantly reduced both blood pressure and sympathetic nerve activity in the hypertensives but not in the normotensive, an effect that was attributed to the improvement in baroreflex autonomic functions [128]. Vitamin C can also target endothelial dysfunction. In one study of 93 human subjects, oral vitamin C (2 g) or its combination with vitamin E (0.6 g) significantly enhanced endothelium-dependent dilatation of the radial artery of patients with coronary artery disease after 2 h [129]. Although the exact molecular mechanism is not clear, increased synthesis and deposition of type IV collagen in the basement membrane, increased endothelial cell proliferation, inhibition of apoptosis and scavenging of free radicals that preserve NO have all been suggested [130]. Dietary vitamin C appears to be even more effective than the supplements as demonstrated by Agrawal et al. [131] who showed that dietary Vitamin C could reduce the progression of carotid artery intima–media thickness while vitamin C supplement could not [131]. The effect of vitamin E alone has also been studied. Milman et al. [132] studied 1434 patients with type 2 diabetes mellitus with haptoglobin 2–2 genotype in a prospective double-blinded clinical trial. The treated group was given 400 IU natural source D-alpha tocopherol once daily for 18 months. The primary outcomes (stroke, CVD death and myocardial infarction) were reduced, with 2.2% for vitamin E versus 4.7% for the placebo group (HR 0.47; 95% CI 0.27 to 0.82; *p* 0.01). This study showed that D-alpha tocopherol may give benefits for patients with high-risk for cardiovascular diseases. Similarly, a relatively low-dose, long-term intervention with vitamin E supplements reduced mortality and atherosclerosis but only when the intervention was started at an early age and taken in a low-cholesterol diet [133]. The protective vascular mechanism was suggested to be the prevention of LDL oxidation which plays an important part in the pathogenesis of atherosclerosis.

Since chronic inflammation can trigger oxidative stress, and oxidative damage can also go on to trigger further inflammation and endothelial dysfunction, the reviewed food plants and their phytochemicals which can both counter inflammation and protect against oxidative damage appear particularly promising for CVD prevention and treatment.

## 7. Conclusions

Redox dysregulation and vascular inflammation play a central role in the development and progression of CVD. For this reason, a multitherapeutic approach which addresses oxidative stress and inflammation is needed for their effective management. The evidence summarized in this review clearly indicates that phytochemicals can play a significant part in counteracting these pathophysiological processes through multiple complementary mechanisms, including direct antioxidant activity, modulation of endogenous defense systems, activation of Nrf2-dependent pathways, and modulation of inflammatory responses. Their cardiovascular relevance is further supported by their effects on endothelial function and vascular homeostasis. In addition to limiting oxidative damage, phytochemicals may help maintain physiological redox signaling, which is essential for vascular homeostasis. However, future studies should also address important translational challenges, including bioavailability, gut microbiota-related metabolism, standardization of phytochemical-rich preparations, and dose optimization. Further mechanistic and clinical studies are needed to quantify and fully harness the benefits, including phytochemicals in the therapeutic strategy for the prevention and treatment of cardiovascular diseases, particularly through well-designed studies with clinically relevant cardiovascular endpoints.

Overall, phytochemicals represent promising modulators of cardiovascular redox homeostasis, but further mechanistic and translational research is required to facilitate their effective integration into cardiovascular health strategies.

## Figures and Tables

**Table 1:** Representative compounds, dietary sources, and key features of flavonoids, non-flavonoids and carotenoids.

	Subclass	Representative Compounds	Primary Dietary Sources	Key Biological/Functional Notes	Ref.
**Flavonoids**	Flavanones	Naringenin, Hesperidin	Citrus fruits	Antioxidant activity; vascular protection	[15,16]
	Isoflavones	Genistein, Daidzein	Soy and other legumes	Phytoestrogenic activity; metabolic and vascular modulation	[17]
	Flavonols	Quercetin, Kaempferol	Onions, apples, kale	Potent Nrf2 modulators; anti-inflammatory properties	[18,19]
	Flavan-3-ols	Epigallocatechin-3-Gallate, Procyanidins	Green/black tea, cocoa, grapes	Strong association with improved FMD and blood pressure	[20]
	Anthocyanins	Cyanidin, Delphinidin	Berries, red wine, purple vegetables	Reduce systemic inflammation; improve endothelial function	[21]
**Non-Flavonoids**	Phenolic Acids	Ferulic acid, Caffeic acid	Whole grains, fruits, coffee	Potent dietary antioxidant	[22,23]
	Stilbenes	Resveratrol	Red grapes, red wine	Modulates redox signaling, longevity pathways, and sirtuins	[24,25]
	Lignans	Secoisolariciresinol, Matairesinol	Flaxseed, sesame, whole grains	Microbiota-derived enterolignans exert cardioprotective and phytoestrogenic effects	[26]
**Carotenoids**	Carotens	β-Carotene, Lycopene	Carrots, tomatoes, sweet potato	Potent antioxidant compounds; reduce oxidative stress and may support cardiovascular health	[27]
	Xanthophylls	Lutein, Zeaxanthin, Astaxanthin	Leafy greens, corn, egg yolk, algae/seafood	Anti-inflammatory and antioxidant activity; associated with endothelial and vascular protection	[28]

**Table 2:** Flavonoid-modulated kinase pathways involved in Nrf2 regulation.

Kinase/Pathway	Flavonoids	Effect on Nrf2	Refs.
PKC/p62	Baicalein	Releases Nrf2 from Keap1; nuclear translocation	[55]
AMPK	Quercetin, Genistein	Phosphorylation enhances nuclear translocation	[55,56]
MAPKs (ERK, p38)	Procyanidin B2, Norartocarpin	Stabilizes Nrf2; activates antioxidant gene transcription	[57]
PI3K/AKT	Quercetin, Genistein	Prevents Nrf2 ubiquitination; enhances stability	[55,56]
PERK	Norartocarpin	Contributes to Nrf2 activation	[56]

Abbreviations: AKT, Protein Kinase B; AMPK, AMP-activated protein kinase; ERK, extracellular signal-regulated kinase; MAPK, mitogen-activated protein kinase; Nrf2, nuclear factor erythroid 2-related factor 2; PERK, protein kinase R-like endoplasmic reticulum kinase; PI3K, phosphoinositide-3-kinase; PKC, protein kinase C.

**Table 3:** Phytochemicals and the reported mode of anti-inflammatory activities.

Compound	Mode of Anti-Inflammatory Activity	Reference
Resveratrol	Nrf2 activation NF-κB inhibition TNF-α, IL-6, IL-1β, NO and ROS Reduction miR-155, miR-21 and miR-146a downregulation	[4,69,70,71,72]
Quercetin	An Nrf2 activation NF-κB inhibition COX-2 and iNOS inhibition miR-155 downregulation	[67,74,75,76]
Curcumin	Nrf2 activation NF-κB inhibition lipid peroxidation inhibition miR-155 downregulation	[77,78,79,80]
Genistein	Nrf2 activation NF-κB inhibition IL-6 and ICAM-1 downregulation miR-155 downregulation	[81,82]
Epigallocatechin-3-Gallate	An Nrf2 activation NF-κB inhibition	[83]
Apigenin	COX-2 inhibition NF-κB inhibition TLR4 downregulation miR-155 downregulation	[4,86,87]
Anthocyanins	Nrf2 activation NF-κB TLR4 downregulation	[88]
Ellagic acid	Nrf2 activation NF-κB inhibition TLR4 inhibition	[89]
Tanshinone IIA	Nrf2 activation miR-155 downregulation	[90,91]
Carvacrol	Nrf2 activation miR-155 downregulation	[90,91]
Thymol	Nrf2 activation miR-155 downregulation	[90,91]
Boswellic acids	Nrf2 activation miR-155 downregulation	[90,91]
Sulforaphane	Nrf2 activation NF-κB inhibition	[92]

## Data Availability

No new data were created in this study.

## Abbreviations

The following abbreviations are used in this manuscript:

AKT	Protein Kinase B
AMPK	AMP-activated protein kinase
CoQ10	Coenzyme Q10
COX-2	cyclooxygenase-2
CVD	Cardiovascular disease
ERK	Extracellular Signal-Regulated Kinase
HDL	high-density lipoprotein
HO-1	heme oxygenase-1
IL-1	interleukin-1
IL-6	interleukin-6
Keap1	Kelch-like ECH-associated protein 1
LDL	low-density lipoprotein
MAPK	mitogen-activated protein kinase
miR	micro RNA
NF-κB	Nuclear factor kappa B
NO	nitric oxide
Nrf2	nuclear factor erythroid 2-related factor 2
PERK	protein kinase R-like endoplasmic reticulum kinase
PI3K	phosphoinositide-3-kinase
PKC	protein kinase C
ROS	reactive oxygen species
TLR4	Toll-like receptor 4
TNF-α	tumor necrosis factor-alpha
